# Supplementary material for: Age and Hair Cortisol Levels as Predictors of SARS-CoV-2 Infection
Source: Int J Environ Res Public Health. 2024 Sep 2;21(9):1166. doi: 10.3390/ijerph21091166 (PMC11430878; doi:10.3390/ijerph21091166)
Supplement: Supplementary file 1 [file ijerph-21-01166-s001.zip › ijerph-3058442-supplementary.pdf]

Table S1. Healthcare Worker Status and Body Mass Index Adjusted Associations between HCC SARS-CoV-2 infection using Ln-transformed Cortisol.

	**Odds Ratio OR (CI)**	*p*
Overall		
HCC	1.17 (0.60, 2.28)	0.64
Healthcare Worker Status (ref = No)	0.93 (0.43, 2.01)	0.84
Body Mass Index *	0.97 (0.90, 1.05)	0.41
Age – stratified analyses **		
≤41 years		
HCC	0.59 (0.24, 1.45)	0.26
Healthcare Worker Status (ref = No)	0.98 (0.33, 2.96)	0.97
Body Mass Index *	0.90 (0.78, 1.05)	0.18
>41 years		
HCC	9.09 (1.39, 59.28)	0.021
Healthcare Worker Status (ref = No)	0.56 (0.13, 2.35)	0.43
Body Mass Index *	1.07 (0.93, 1.23)	0.38

* BMI for this analysis uses the numeric BMI values as a covariate; ** Interpretation for odds ratios in this table: When ln-HCC increases by 1, the odds of a positive SARS-COV-2 test for >41 years are 9.09 times the odds before the increase. This translates into an odds ratio of 5.32 when ln-HCC increases by 1 interquartile range (IQR = 0.757).
